# Mouse Grueneberg ganglion neurons share molecular and functional features with *C. elegans* amphid neurons

**DOI:** 10.3389/fnbeh.2013.00193

**Published:** 2013-12-09

**Authors:** Julien Brechbühl, Fabian Moine, Marie-Christine Broillet

**Affiliations:** Department of Pharmacology and Toxicology, Faculty of Biology and Medicine, University of LausanneLausanne, Switzerland

**Keywords:** olfactory, amphid neurons, Grueneberg ganglion, behavior, calcium imaging, temperature sensing, alarm pheromone

## Abstract

The mouse Grueneberg ganglion (GG) is an olfactory subsystem located at the tip of the nose close to the entry of the naris. It comprises neurons that are both sensitive to cold temperature and play an important role in the detection of alarm pheromones (APs). This chemical modality may be essential for species survival. Interestingly, GG neurons display an atypical mammalian olfactory morphology with neurons bearing deeply invaginated cilia mostly covered by ensheathing glial cells. We had previously noticed their morphological resemblance with the chemosensory amphid neurons found in the anterior region of the head of *Caenorhabditis elegans* (*C. elegans*). We demonstrate here further molecular and functional similarities. Thus, we found an orthologous expression of molecular signaling elements that was furthermore restricted to similar specific subcellular localizations. Calcium imaging also revealed a ligand selectivity for the methylated thiazole odorants that amphid neurons are known to detect. Cellular responses from GG neurons evoked by chemical or temperature stimuli were also partially cGMP-dependent. In addition, we found that, although behaviors depending on temperature sensing in the mouse, such as huddling and thermotaxis did not implicate the GG, the thermosensitivity modulated the chemosensitivity at the level of single GG neurons. Thus, the striking similarities with the chemosensory amphid neurons of *C. elegans* conferred to the mouse GG neurons unique multimodal sensory properties.

## Introduction

**Figure 1:**
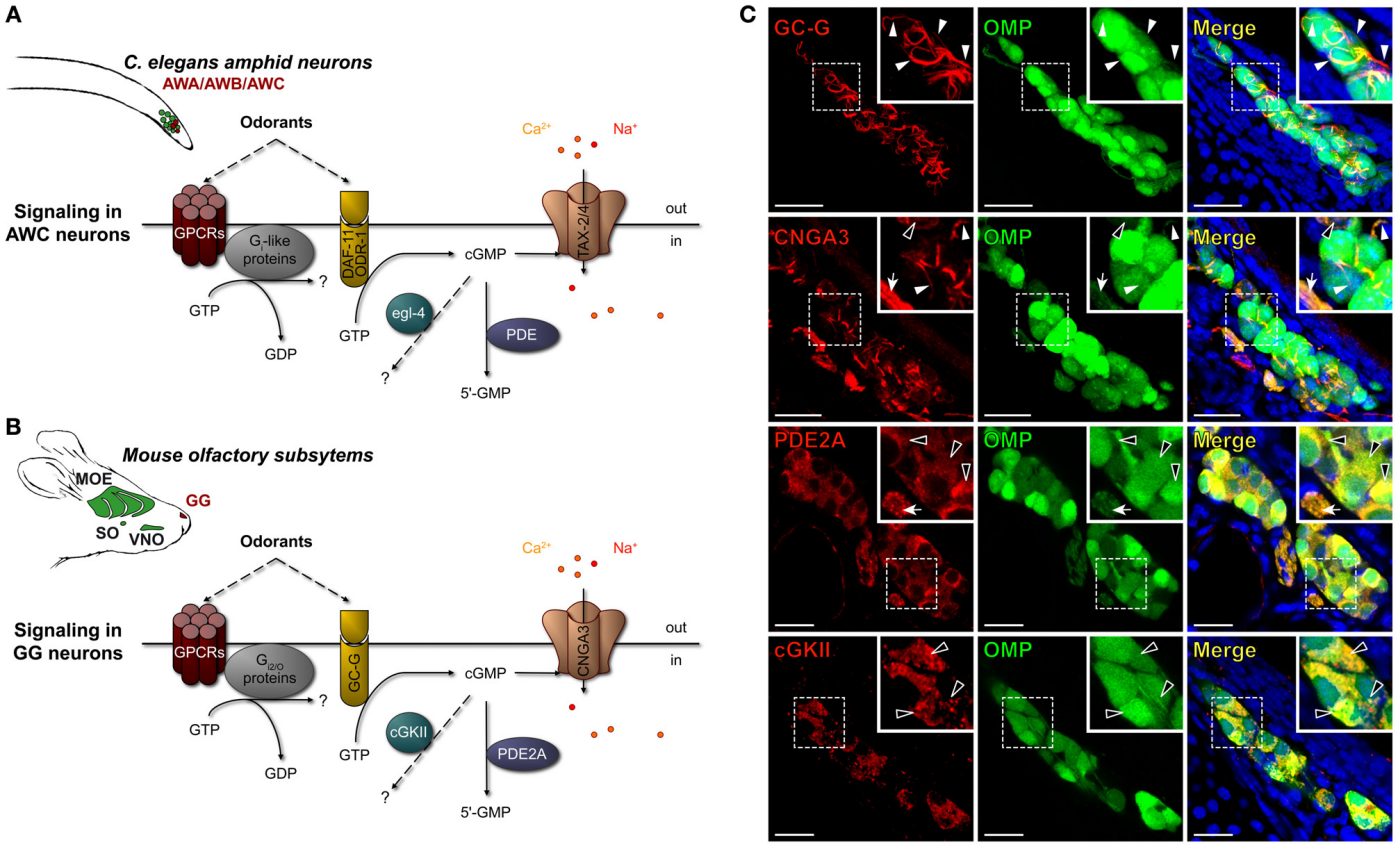
**Mouse GG neurons express in conserved subcellular localizations a set of signaling proteins related to those present in *C. elegans* chemosensory amphid neurons**. **(A,B)** Schematic comparison of the main signaling proteins expressed in *C. elegans* amphid AWC neurons **(A)** and in the mouse GC-G positive GG neurons **(B)**. *C. elegans* amphid neurons are indicated (in green), in particular the AWA, AWB, and AWC neurons (in red). The mouse olfactory subsystems (in green) are indicated as follows: GG, Grueneberg ganglion (in red); VNO, vomeronasal organ; SO, septal organ; MOE, main olfactory epithelium. The signaling elements are indicated as follows: cGMP, cyclic guanosine monophosphate; cGKII, cGMP-dependent protein kinase of type 2; CNGA3, cyclic nucleotide-gated channels 3; DAF-11/ODR-1, potential receptor-like transmembranous guanylyl cyclase; egl-4, cGMP-dependent protein kinase; GC-G, particulate guanylyl cyclase G; GPCRs, G protein coupled receptors; PDE, phosphodiesterase; PDE2A, phosphodiesterase 2A; TAX-2/4, cyclic nucleotide-gated (CNG)-like channels. **(C)** Immunohistochemistry experiments on coronal slices of the GG of OMP-GFP mice for homologous signaling proteins found in *C. elegans* amphid neurons. GC-G was found to be expressed in the cilia. CNGA3 was found to be expressed principally in cilia and axons; some somatic expression could also be observed. PDE2A was found in soma and in axons. cGKII was found in soma. The specific subcellular localizations are shown in high power views (white dashed rectangles). White arrowheads indicate ciliary processes, black arrowheads indicate soma and white arrows indicate axons. A minimum of 2 animals (from P0–P29) and 6 slices were used for each antibody staining tested. Nuclei are shown in blue (DAPI counterstain). Scale bars, 20 μm.

Organisms have evolved specialized populations of sensory receptor neurons to detect the chemical information that is present in their environment (Ache and Young, 2005). In the model organism *Caenorhabditis elegans* (*C. elegans*), chemo- and thermo-sensing are performed by 12 pairs of ciliated sensory neurons, the amphid neurons, that are found in the anterior region of the animal head (Mori and Ohshima, 1995; Bargmann, 2006). Amphid neurons include three principal olfactory classes named amphid wing neurons of type A (AWA), B (AWB), and C (AWC) (Figure 1A). They play a fundamental role in mate identification, in food finding and in noxious conditions avoidance by odortaxis (Bargmann, 2006). They also participate in the development of the organism by navigating through spatial thermal gradients by thermotaxis (Mori and Ohshima, 1995). In the mouse, the olfactory sensory neurons are dispatched in four distinct subsystems: the main olfactory epithelium (MOE), the septal organ of Masera (SO), the vomeronasal organ (VNO) and the most recently described Grueneberg ganglion (GG) (Gruneberg, 1973) (Figure 1B). These subsystems are implicated in the detection of molecules carrying chemical messages, such as odorants and pheromones that play a role in behaviors ranging from food finding to social communication (Munger et al., 2009). We have identified one of these subsystems, the GG, as a chemodetector of alarm pheromones (APs) (Brechbühl et al., 2008). APs signal injury, distress or the presence of predators (Kiyokawa et al., 2004). A wide variety of changes in behaviors can be observed in the presence of APs such as a decreased exploratory activity or increase in vigilance and freezing behaviors (Zalaquett and Thiessen, 1991; Kiyokawa et al., 2006). We have recently isolated and identified the chemical structure of one mouse AP, 2-*sec*-butyl-4,5-dihydrothiazole (SBT) (Brechbühl et al., 2013). This volatile APs is produced by both male and female mice under different alarm conditions and resembles the sulfur-containing volatiles present in predator scents. In addition to its reported chemosensory modality (Brechbühl et al., 2008, 2013; Mamasuew et al., 2011a; Hanke et al., 2013), the mouse GG has also been implicated in sensing cold temperatures (Mamasuew et al., 2008; Schmid et al., 2010).

We noticed the morphological resemblance between the mouse GG neurons and the olfactory AWA, AWB, and AWC amphid neurons from the nematode *C. elegans* (Brechbühl et al., 2008). Indeed, mouse GG neurons display an atypical olfactory morphology with neurons that bear deeply invaginated cilia and that are mostly covered by glial cells (Gruneberg, 1973; Tachibana et al., 1990; Brechbühl et al., 2008; Liu et al., 2009). They have a rostral localization in a water permeant epithelium and they lack direct contact with the nasal cavity (Gruneberg, 1973; Fuss et al., 2005; Koos and Fraser, 2005; Fleischer et al., 2006a; Roppolo et al., 2006; Storan and Key, 2006; Brechbühl et al., 2008; Liu et al., 2009). In *C. elegans*, pairs of either AWA, AWB, or AWC neurons detect volatile odorants. They are found under a water permeant cuticle and are wrapped by a single ensheathing glial cell that also surrounds the modified cilia (Bargmann et al., 1993; Bargmann, 2006; Inglis et al., 2007; Bacaj et al., 2008).

These morphological similarities might also indicate molecular similarities between mouse GG neurons and *C. elegans* amphid neurons. Indeed, recent studies have revealed that canonical and non-canonical signaling elements are expressed in GG neurons (Fleischer et al., 2006b, 2007, 2009; Pyrski et al., 2007; Brechbühl et al., 2008; Liu et al., 2009, 2012). They could, as in *C. elegans*, be potentially implicated in parallel and/or convergent G protein-coupled receptors (GPCRs)- and cyclic guanosine monophosphate (cGMP)-dependent signaling pathways for thermo- and chemodetection (Mamasuew et al., 2010, 2011a,b; Schmid et al., 2010; Hanke et al., 2013).

Here, we investigated the conserved multisensory modalities of mouse GG neurons. We found striking similarities between mouse GG neurons and nematode amphid neurons, especially the AWC class. We found that 2,4,5-trimethylthiazole, a known AWC ligand, was able to initiate neuronal responses in mouse GG neurons in a cGMP-dependent manner. AWC neurons are also known to act as thermosensors and we found here that the temperature can modulate the chemosensitivity of GG neurons. Thus, GG neurons, through their position at the tip of the nose, are able to integrate multiple sensory inputs thereby allowing an animal to assess additional aspects of its olfactory environment.

## Experimental procedures

### Animals and tissue preparation

OMP-GFP mice (Potter et al., 2001) from pups to adult stages were used for all experimental investigations. This particular gene-targeted mouse strain expresses the green fluorescent protein (GFP) as a histological reporter under the control of the olfactory marker protein (OMP) promoter (Mombaerts et al., 1996; Potter et al., 2001). OMP is a marker specific for mature olfactory sensory neurons (Margolis, 1972). Animal care was in accordance with the Swiss legislation and the veterinary authority. Mice were killed by CO_2_ or cervical dislocation. Nasal cavities were prepared in ice-cold artificial cerebrospinal fluid (ACSF), containing 118 mM NaCl, 25 mM NaHCO_3_, 10 mM D-glucose, 2 mM KCl, 2 mM MgCl_2_, 1.2 mM NaH_2_PO_4_, and 2 mM CaCl_2_ (pH 7.4) saturated with oxycarbon gas [95% O_2_: 5% CO_2_; (vol/vol)] under a fluorescence-equipped dissecting microscope (M165 FC; Leica). For specific experiments, ACSF calcium free solution was also used and it was composed of NaCl 118 mM, NaHCO_3_ 25 mM, D-Glucose 10 mM, KCl 2 mM, MgCl_2_ 2 mM, NaH_2_PO_4_ 1.2 mM, EDTA 10 mM and EGTA 10 mM saturated with oxycarbon gas.

### Immunohistochemistry

Protocol for floating immunohistochemistry was adapted from (Brechbühl et al., 2011, 2013). Briefly, the tip of the nose was carefully dissected in PBS (138 mM NaCl, 2.7 mM KCl, 0.9 mM KH_2_PO_4_, and 0.8 mM Na_2_HPO_4_, pH 7.6) before being fixed in 4% paraformaldehyde (PAF 4%, in PBS pH 7.4; 158127, Sigma) at 4°C for 3 h. Fixed tissue preparations were embedded in 5% agar (A7002, Sigma) prepared in PBS. Agar blocks were transferred on ice for 30 s for solidification. The agar blocks were fixed vertically with cyanacrylat glue (Roti coll 1, Carl Roth) onto the holder of the vibroslicer (VT1200S, Leica). 80 μm coronal sections were cut in PBS and were selected with a fluorescence-equipped dissecting microscope (M165 FC; Leica). Slices were blocked overnight at 4°C in a PBS solution containing 10% NGS (normal goat serum, Jackson ImmunoResearch) and Triton X-100 0.5%. Primary antibodies were applied to the slices for 16 h at RT in a PBS solution containing NGS 5% and Triton X-100 0.25%. Slices were washed in NGS 2% and were incubated in the dark with the secondary antibody in a PBS solution containing NGS 2% for 1 h at RT. Slices were finally washed and mounted in Vectashield (H-1200, Vector Labs) with DAPI mounting medium. The primary antibodies used for the detection of signaling proteins were the particulate guanylyl cyclase G [GC-G (PGCG-701AP); 1:300, Rabbit, FabGennix], the cyclic nucleotide-gated channel type 3 [CNGA3 (LS-C14509); 1:300, Rabbit, Lifespan Bioscience], the phosphodiesterase 2A [PDE2A (PD2A-101AP); 1:500, Rabbit, FabGennix] and the cGMP-dependent protein kinase type II [cGKII (H-120, sc-25430); 1:50, Rabbit, Santa Cruz biotechnology]. The secondary antibody used was coupled to Cy3 [Cy3-conjugated AffiniPure anti-Rabbit (111-165-144); 1:200, Goat, Jackson ImmunoResearch]. Control experiments were performed by omitting primary antibodies. Observations and acquisitions were made by confocal microscopy (SP5, Leica) under objectives of 40–100×. Post-analysis and reconstructions were made with Imaris (Bitplane IMARIS 6.3).

### Protein sequence analyses

The protein sequences were obtained from the National Centre for Biotechnology Information (NCBI) database. We chose the following *C. elegans* sequences DAF-11 (gi: 198447220); TAX-4 (gi: 13548488); pde-2 (gi: 71989276); egl-4 (gi: 71989393) and obtained, respectively, the following mouse homologous sequences after BLASTP algorithm: GC-G (gi: 124487301); CNGA3 (gi: 530537234); PDE2A (gi: 344217717); cGKII (gi: 188219585). Scores of identity and similarity were used to evaluate the sequence homologies.

### Calcium imaging

Calcium imaging experiments were performed on acute tissue slices of mouse GG (Brechbühl et al., 2008, 2013). Briefly, pups and adult mice were killed and dissected in fresh ACSF solution at 4°C. The tip of the mouse nose was included in a block of low melting 5% agar at 41°C and directly placed on ice for solidification. Blocks were fixed vertically with cyanacrylat glue (Roti coll 1, Carl Roth) on the object holder and coronal slices from 60–80 μm were cut at 4°C with a vibroslicer (VT1200S, Leica). Multiple slices could be obtained from one mouse GG. Slices were selected for their GFP expression with a fluorescent stereomicroscope (M165 FC, Leica). Selected slices were loaded with Fura-2 acetoxymethyl ester (AM) (5 μ M; TEFLabs) and pluronic acid (0.1%; Pluronic F-127, Invitrogen) in an incubator for 45 min (37°C, 5% CO_2_). Slices were placed in the bath chamber (RC-26, Warner Instruments) and immobilized with a slice anchor. Observations were made under an inverted fluorescence microscope (Axiovert 135, Zeiss) with a 25 or 63x objective and Cool SNA-HQ camera. A bipolar temperature controller (SC-20/CL-100, Warner instruments) was used to control the bath temperature. RT (room temperature) corresponded to 23–25°C. The software MetaFluor (MetaFluor, Visitron Systems) was used to monitor intracellular calcium and to acquire images (Brechbühl et al., 2011).

### Chemostimulation

Odorant and drugs were obtained from Sigma-Aldrich and were prepared freshly before each experiment by direct dilution in ACSF. For pyrazine, a stock solution was prepared in alcohol (w:v; 1:2) before final dilution (Bargmann et al., 1993; Tonkin et al., 2002). The osmolarity of solutions was between 285 and 300 Osm/L. These chemical cues are known to reversibly mediate intracellular calcium increases in *C. elegans* when used at 1:1,000–1:1,000,000 dilutions (Lans et al., 2004; Chalasani et al., 2007). For this reason, for each tested cue, a range of concentrations from 100–1 μ M was used in our experiments. A short exposure to an extracellular potassium concentration of 20 mM was used as a viability test and standard reference. The percentages of responses were standardized by comparing the calcium increases observed with KCl vs. the ones observed with the tested cue. Fura-2AM ratio (F340/380 nm) observed during the perfusion of ACSF was considered as baseline activity; it corresponded to ~5% of a KCl response. Calcium increases twice larger than this baseline activity (10% of the KCl response) were considered as responses (Brechbühl et al., 2013). 8-bromoguanosine 3′,5′-cyclic monophosphate (8-Br; 500 μM) was used to mimic the intracellular source of cGMP in GG (Schmid et al., 2010). To inhibit CNGA3 channels, the CNG inhibitor L-*cis* diltiazem hydrochloride (Dilt) was used (100–500 μM) and continuously perfused on tissue slices during a minimum of 5 min before perfusing any tested cues for inhibition tests (Frings et al., 1992). In calcium free experiments, the ACSF calcium free solution was perfused during a minimum 5 min before any cues were perfused.

### Thermotaxis and huddling

A total of 31 pups from OMP-GFP mice from 5 different litters were used to study thermotaxis and huddling behaviors. As previously described (Roppolo et al., 2006; Brechbühl et al., 2008), surgical ablation of the GG was performed in 25 P0 mice by cutting the GG axon bundles. Untreated mice did not undergo the surgical procedure (*n* = 6). In order to assess the effective surgical procedure, mice were phenotyped at P15 (after the behavioral sessions), using a stereomicroscope (MZ16FA, Leica). GFP fluorescence of the GG and, as control, the MOE was observed. Mice were considered as axotomized mice (Axo; *n* = 18/25) in case of total absence of GFP fluorescence at the normal localization of the GG, the other mice were considered as sham control mice (Ctrl; *n* = 7/25). Between behavioral tests, pups were returned to their mothers and littermates. By precaution, adult male mice were kept separate from the litters. Because no differences were measured between untreated and Ctrl mice, only results from Ctrl and Axo mice are presented for clarity purposes. The thermotaxis protocol was adapted from (Pacheco-Cobos et al., 2003; Serra and Nowak, 2008). Untreated, Ctrl and Axo mice were placed at P5, P9, and P12 in a Plexiglas arena (13 × 13 cm) in which a thermal gradient (37–0°C) was generated by controlling the temperatures of the walls. To prevent place preference, a four-session test design was performed for each pup, where the temperatures of the walls changed clockwise. Between each session, the Plexiglas arena was cleaned with alcohol and water. Sessions of 3 min were recorded with a standard HD camera and/or a thermal camera (ThermaCAM™ E45, FLIR Systems). Post-analysis was performed with computer assistance, the position of the body center (green dots) as well as the tip of the nose (red dots, corresponding to the GG region) were reported as a function of time (*t* = 0s, *t* = 30s, *t* = 60s, *t* = 120s) and the first contact of the nose with the hottest wall was measured. To determine the huddling behavior, six pups (at P5, P9, and P12) were placed in the center of a Plexiglas arena (13 × 13 cm) at RT (23–25°C). Sessions of 3 min were recorded with a standard HD camera and/or a thermal camera (ThermaCAM™ E45, FLIR Systems). Active behaviors (climbing, contact maintenance) as well as body temperature were measured.

### Statistics

For statistical comparisons, open source statistical package R version 3.0.2 was used. Normality and homogeneity were evaluated by the Shapiro test. Monofactorial comparisons were done with student's *t*-tests or Wilcoxon *w*-tests. Multifactorial comparisons were done by ANOVA. Values are expressed as mean ± s.e.m. Significance levels are indicated as follows: ^*^p < 0.05; ^**^p < 0.01; ^***^p < 0.001; ns for non-significant.

## Results

### Conserved molecular signaling in mouse GG neurons

The amphid AWA, AWB, and AWC neurons of *C. elegans* express multiple molecular signaling proteins that are directly related to their functional roles (Bargmann, 2006). For example, in a single AWC neuron, one can find, in specific subcellular localizations, both canonical and non-canonical GPCRs (Sengupta et al., 1996; Battu et al., 2003; Alcedo and Kenyon, 2004), potential receptor-like transmembranous guanylyl cyclase DAF-11/ODR-1 (Vowels and Thomas, 1994; Birnby et al., 2000), downstream elements G_*i*_-like proteins (Roayaie et al., 1998; Jansen et al., 1999), phosphodiesterases (PDE) (Bargmann, 2006; O'halloran et al., 2012), cGMP-dependent protein kinase (egl-4) (L'etoile et al., 2002; O'halloran et al., 2009; Lee et al., 2010) and cyclic nucleotide-gated (CNG)-like channels TAX-2/4 (L'etoile and Bargmann, 2000; Kaupp and Seifert, 2002; Bargmann, 2006). Interestingly, similar signaling proteins are also present in mouse GG neurons (Fleischer et al., 2006b, 2007, 2009; Brechbühl et al., 2008; Liu et al., 2009) (Figures 1A,B). In a first approach, we focused on the cGMP-dependent proteins and verified by immunohistochemistry their neuronal expression and localization in mouse GG neurons (Figure 1C). GG tissue slices were obtained from OMP-GFP transgenic mice (Mombaerts et al., 1996; Potter et al., 2001). We looked for the presence of the particulate guanylyl cyclase G (GC-G), a potential transmembrane receptor (Fleischer et al., 2009; Liu et al., 2009), the cyclic nucleotide-gated channels 3 (CNGA3) (Liu et al., 2009) as well as downstream regulatory elements such as the phosphodiesterase 2A (PDE2A) (Fleischer et al., 2009; Liu et al., 2009; Matsuo et al., 2012) and the cGMP-dependent protein kinase of type 2 (cGKII) (Liu et al., 2009). We found GC-G exclusively in ciliary structures but CNGA3 in cilia, cell bodies and axons. The downstream element PDE2A was expressed in cell bodies and axons but cGKII was located exclusively in the cell bodies. These specific subcellular localizations were conserved among GG neurons and mice (>90 neurons were checked in each GG slice corresponding to >1000 neurons observed for the expression of each investigated proteins). Moreover, these expression patterns were identical to those found in amphid neurons (Coburn and Bargmann, 1996; Mccleskey, 1997; Coburn et al., 1998; Dwyer et al., 1998; Bargmann, 2006). We next evaluated, by BLAST algorithm, the identity and similarity of these GG-expressed proteins with the ones of amphid neurons. We found that the mouse GC-G shares 29% identity and 65% similarity with the *C. elegans* DAF-11, CNGA3 and TAX-4 share 44% identity and 91% similarity, PDE2A and pde-2 share 38% identity and 58% similarity and cGKII and egl-4 share 47% identity and 96% similarity. The similar expression patterns as well as their high similarity scores suggest that these molecular signaling elements might be an orthologous set of proteins (Altenhoff and Dessimoz, 2009).

### Conserved chemosensitivity of mouse GG neurons

**Figure 2:**
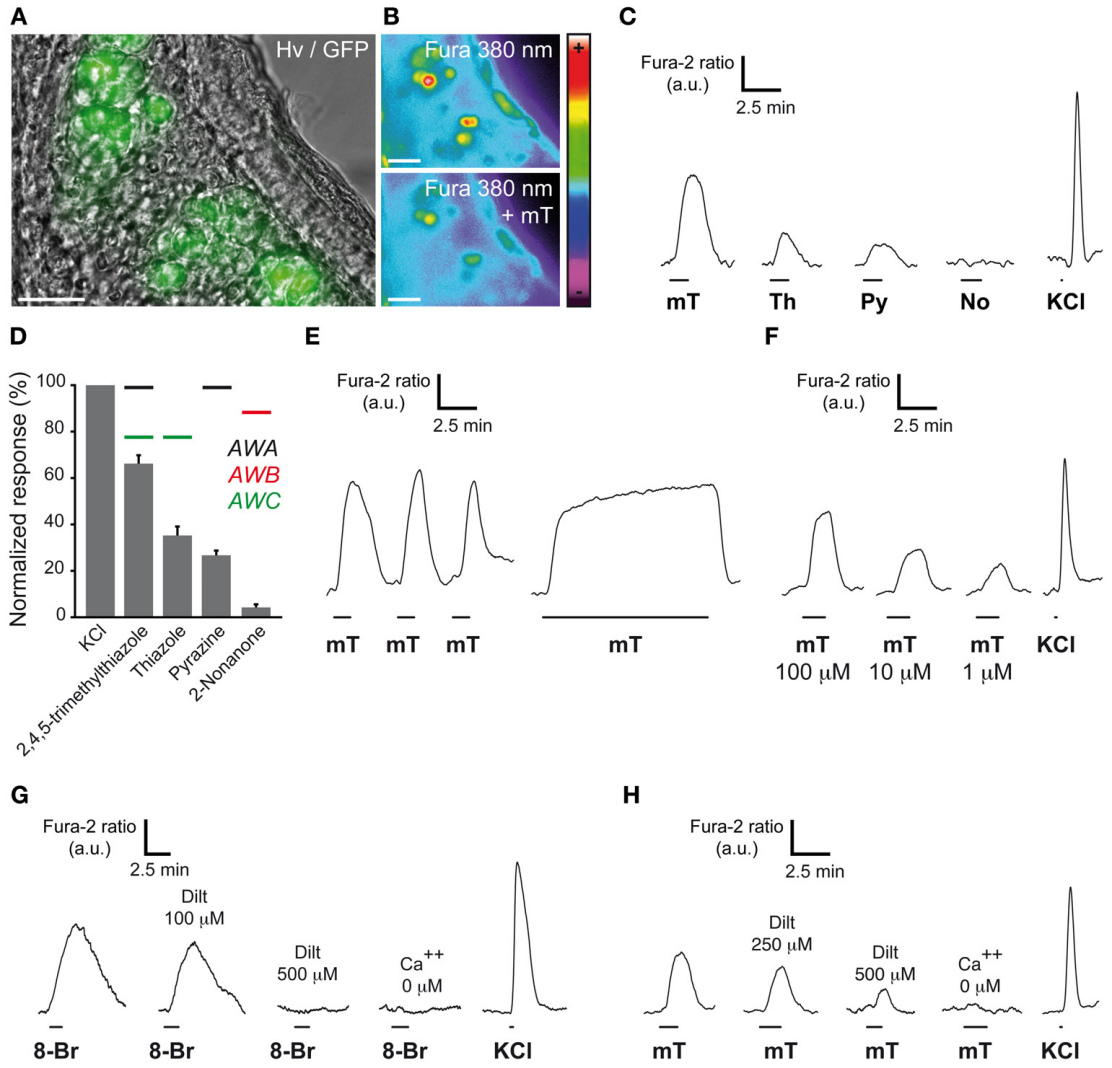
**GG neurons respond to ligands of amphid neurons. (A)** GG coronal slice from an OMP-GFP mouse where GG neurons can be observed with their intrinsic GFP fluorescence and Hoffman modulation view (Hv). **(B)** Fluorescence of Fura-2AM into GG cells observed at 380 nm in color encoded map for unbound Fura before and at the peak of an intracellular calcium increase induced by 2,4,5-trimethylthiazole (mT). **(C–H)** Chemostimulations of GG neurons realized at RT. **(C)** Representative calcium transients induced in the same GG neuron by the successive perfusion of amphid AWA, AWB, and AWC ligands (100 μ M). **(D)** Histogram summarizing Fura-2 ratio peak Ca^2+^ responses to stimulation with the different activators (100 μ M) normalized to KCl responses (20 mM). Perfusion of mT (*n* number of responding neurons/number of total neurons = 66/66), thiazole (Th; *n* = 14/16), pyrazine (Py; *n* = 32/34) but not 2-nonanone (No; *n* = 0/18) increased the intracellular calcium concentration. Color bars indicate the AWA (in black), AWB (in red) or AWC (in green) ligand's relationship. 2–9 mice (P1–P26) were used for each tested chemical. Values are expressed as mean ± s.e.m. **(E)** mT responses were rapidly reversible and reproducible (*n* number of tested neurons = 16). No adaptation was observed in the presence of mT for a period of 10 min (*n* = 15). **(F)** The calcium increases generated by 2,4,5-trimethylthiazole (mT) were observed over a broad concentration range (from 100–1 μ M; *n* = 10). **(G)** Representative calcium transients induced in GG neurons by perfusion of a cGMP membrane-permeable analog, the 8-bromoguanosine 3′, 5′-cyclic monophosphate (8-Br; 500 μ M), and inhibited by increasing concentrations of the cyclic nucleotide-gated channel blocker L-*cis* diltiazem (Dilt, *n* = 11). A 8-Br response was not observed in calcium-free medium (*n* = 48). **(H)** mT responses could be partially inhibited by L-*cis* diltiazem [Dilt 250 μ M (26%, *n* = 11); 500 μ M (58%, *n* = 6)]. Fluorescence intensity Fura-2 ratio = F340/F380 is indicated by arbitrary units (a.u.). Perfusion times are indicated by horizontal bars. Traces illustrated in **(C)**, **(E)**, **(F)**, **(G)**, and **(H)** are representative responses observed in single GG neurons for each panel. Scale bars, 20 μm in **(A)** and **(B)**.

In amphid neurons, these cGMP-related proteins participate in chemosensing (Bargmann, 2006). Indeed, volatile water soluble cues such as pyrazine for AWA neurons, 2-nonanone for AWB neurons, thiazole and 2,4,5-trimethylthiazole for AWC neurons (Bargmann, 2006) are known to reversibly induce intracellular calcium increases. This neuronal stimulation is partially mediated by the second messenger cGMP (Coburn et al., 1998; Bargmann, 2006). To verify if these known ligands of amphid neurons could also activate GG neurons, we performed calcium imaging experiments on GG coronal slices from OMP-GFP mice (Brechbühl et al., 2008). Tissue slices were incubated in Fura-2AM, a ratiometric calcium-sensitive dye. GG cells were identified by the intrinsic green fluorescence of GFP in their cell bodies and by their specific morphology (Figure 2A). The uptake of the dye was confirmed by fluorescence observations (Figure 2B). Chemical stimuli were delivered at room temperature in oxycarbonated ACSF continuously perfused on the tissue slices in the imaging chamber and the neuronal viability was evaluated by a brief stimulation of KCl (Figures 2B,C). We found that stimulation with these ligands of amphid neurons evoked calcium transients of different amplitudes in most mouse GG neurons. Interestingly, the AWA and AWC ligand 2,4,5-trimethylthiazole induced the largest responses (mT; 66.2 ± 3.6%; *n* = 66 responding neurons/66 tested neurons) (Figure 2D). Smaller responses were observed with thiazole (Th; 35.2 ± 3.9%; *n* = 14/16) and pyrazine (Py; 26.7 ± 2.0%; *n* = 32/34). Single GG neurons could be stimulated by all tested AWA and AWC ligands (Figure 2C; *n* = 14/14). On the other hand, in responding neurons no activation was observed with the AWB ligand 2-nonanone (No; 4.2 ± 1.3%; *n* = 0/18), thus conferring to GG neurons a selectivity for related-chemical structures (Brechbühl et al., 2013). Calcium increases due to mT were rapidly reversible and reproducible (Figure 2E). No adaptation was observed in the presence of the stimulus for a period of 10 min (Figure 2E). Responses occurred over a broad range of concentrations (tested from 100–1 μ M) (Figure 2F). Recently, dependence of GG chemosensitivity on cGMP signaling has been shown using animals lacking cGMP-associated signaling proteins (Mamasuew et al., 2011b; Hanke et al., 2013). The calcium transients we observed could be mimicked by perfusion of the cGMP membrane-permeable analog 8-bromoguanosine 3′, 5′-cyclic monophosphate (8-Br; 500 μM; *n* = 88/95) (Zufall and Munger, 2010) (Figure 2G). Thus, in addition to previous reports, we showed that the cyclic nucleotide-gated channel blocker L-*cis* Diltiazem (Dilt; 500 μM) was able to inhibit completely the calcium transients generated by 8-Br (Figure 2G; *n* = 11/11) but only partially those generated by mT (58%) (Figure 2H; *n* = 6/6). The presence of extracellular calcium was necessary to observe an 8-Br or mT response (Figures 2G,H). It therefore appears that mouse GG neurons also partially display a cGMP-dependent chemosensitivity resembling the one of AWC amphid neurons (Coburn et al., 1998; Fujiwara et al., 2002; Bargmann, 2006; Tsunozaki et al., 2008).

### Temperature-dependent chemosensitivity of GG neurons

**Figure 3:**
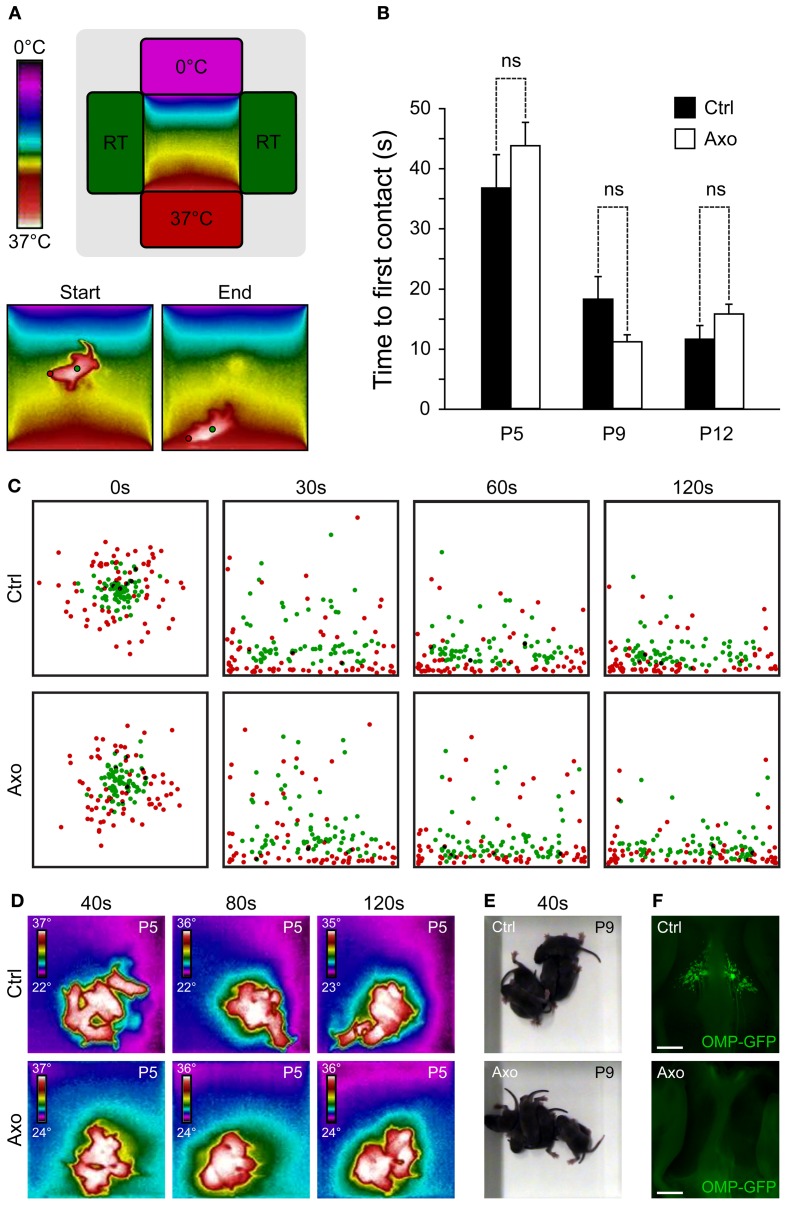
**A functional GG is not necessary for thermotaxis and huddling behaviors**. **(A–C)** Thermotaxis analysis. **(A)** Schematic representation of the arena visualized with a thermal camera. The pup is placed in the middle of the arena and, during a session of 3 min, localization is recorded. Red dot represents the GG localization and, the green dot, the body center. **(B)** The time necessary for the tip of the nose (red dot) to be in contact with the 37°C wall is measured, for P5, P9, and P12. Experiments were done with sham control (Ctrl; *n* = 7) pups and GG axotomized (Axo; *n* = 18) pups; each pup has been used in 4 sessions. **(C)** Merge view of the localization of the tip of the nose (red dots) and body center (green dots) after 0, 30, 60, and 120 s for P5, P9, and P12. For clarity, only the 4 sessions of 6 different pups (at the three tested ages) per phenotype were plotted **(C)**. **(D–F)** Huddling analysis. **(D)** Thermal view of 6 pups per phenotype at P5, after 40, 80, and 120 s. **(E)** Normal view at P9 after 40 s. **(F)** Phenotyping was done at the end of the thermotaxis and huddling behavioral sessions, at P15. The Ctrl (presence of GG) and Axo (no GG) mice were grouped for post-analysis. Scale bars, 500 μm in **(F)**. Values are expressed as mean ± s.e.m.; *w*-test, ^*^P < 0.05; ^**^P < 0.01; ^***^P < 0.001; ns, not significant.

Interestingly, the chemosensitive amphid neurons, especially the AWC neurons, are also sensitive to temperature variations, which influence animal behavior (Biron et al., 2008; Kuhara et al., 2008). Indeed, genetic or laser deletion of AWC neurons, or members of their signaling pathway, demonstrated their contribution to the animal thermotactic behavior (Biron et al., 2008). Mouse GG neurons are also known to be sensitive to temperature changes (Mamasuew et al., 2008; Schmid et al., 2010) and in mice, two essential behaviors depend on temperature sensing, thermotaxis and huddling (Pacheco-Cobos et al., 2003; Alberts, 2007). We therefore tested the potential role of the GG in these two behaviors, comparing sham control mice (Ctrl) with GG axotomized mice (Axo) (Roppolo et al., 2006; Brechbühl et al., 2008, 2013). We focused on mouse pups, as the maintenance of body temperature within narrow limits is one of their most basic homeostatic needs (Pacheco-Cobos et al., 2003). Contrary to their homoiothermic mother, mouse pups are poikilothermic, which means that their body temperature varies with the temperature of their surroundings (Alberts, 2007). When mouse pups are separated from their mother, their internal temperature drops. Thus, when pups are placed in a thermal gradient, they naturally search by active movements the warmest region (Pacheco-Cobos et al., 2003). To evaluate the potential implication of the GG in mouse thermotaxis, we used a behavioral arena where a thermal gradient was generated and we filmed the pups performance with a combination of a normal and a thermal camera (Figure 3A). Performance was assessed in Ctrl (*n* = 7) and Axo (*n* = 18) animals of different ages by placing the pups in the middle of the arena and subsequently measuring the time of the first contact with the 37°C wall at different ages (Figure 3B). As expected, age was a critical factor for the mice performance (ANOVA: ^***^), but phenotype was not relevant (ANOVA: ns). Indeed, no significant differences were observed between Ctrl and Axo pups at P5 (Ctrl: 36.7 ± 5.5 s; Axo: 43.7 ± 3.9 s; *w*-test: ns), P9 (Ctrl: 18.2 ± 3.7 s; Axo: 11.1 ± 1.2 s; *w*-test: ns) nor at P12 (Ctrl: 11.5 ± 2.3 s; Axo: 15.7 ± 1.7 s; *w*-test: ns). Temporal localizations of the tip of the nose (corresponding to the GG location) as well as the body center were plotted (Figure 3C and supplementary Movies 1, 2), which demonstrated that both phenotypes were equally efficient in thermotaxis. Indeed, after 30 s the majority of the pups had found their final position. Furthermore, for both Ctrl and Axo mice, the tip of the nose was the body part that was found physically closest to the 37°C wall.

In addition to thermotaxis, mouse pups have to develop efficient huddling behaviors (Alberts, 2007). Huddles of pups are aggregations established by the tendency of pups to approach one another and then actively maintain contact. By huddling, mouse pups are able to preserve their body heat and thus reduce their metabolic expenditure in relation to the ambient temperature, saving their energy for growth (Alberts, 2007). Pups within a huddle are observed to root and burrow between other bodies, climb on one another, crawl around the periphery and then re-enter the huddle (Schank and Alberts, 1997). A multitude of sensory cues, including tactile, thermal and olfactory stimuli, govern the behavior of pups (Alberts, 1978). We evaluated the huddling behaviors of Ctrl (*n* = 6) and Axo (*n* = 6) mice pups (at P5 and P9) placed in the middle of an arena at room temperature and recorded for 3 min (Figures 3D,E). Both phenotypes were active and typical features of huddling as “climbing” and “contact maintenance” were observed. In addition, the body temperatures as well as the size of the “mice aggregates” were similar between both groups (supplementary Movies 3, 4). Thus, the absence of a functional GG did not seem to affect the natural huddling behavior of mice pups. The evaluation of the phenotype was done by stereomicroscopy observations after the thermotaxis and huddling behavioral sessions at P15 (Figure 3F). Based on these results, these two behaviors are not governed by thermal sensors expressed in GG neurons.

**Figure 4:**
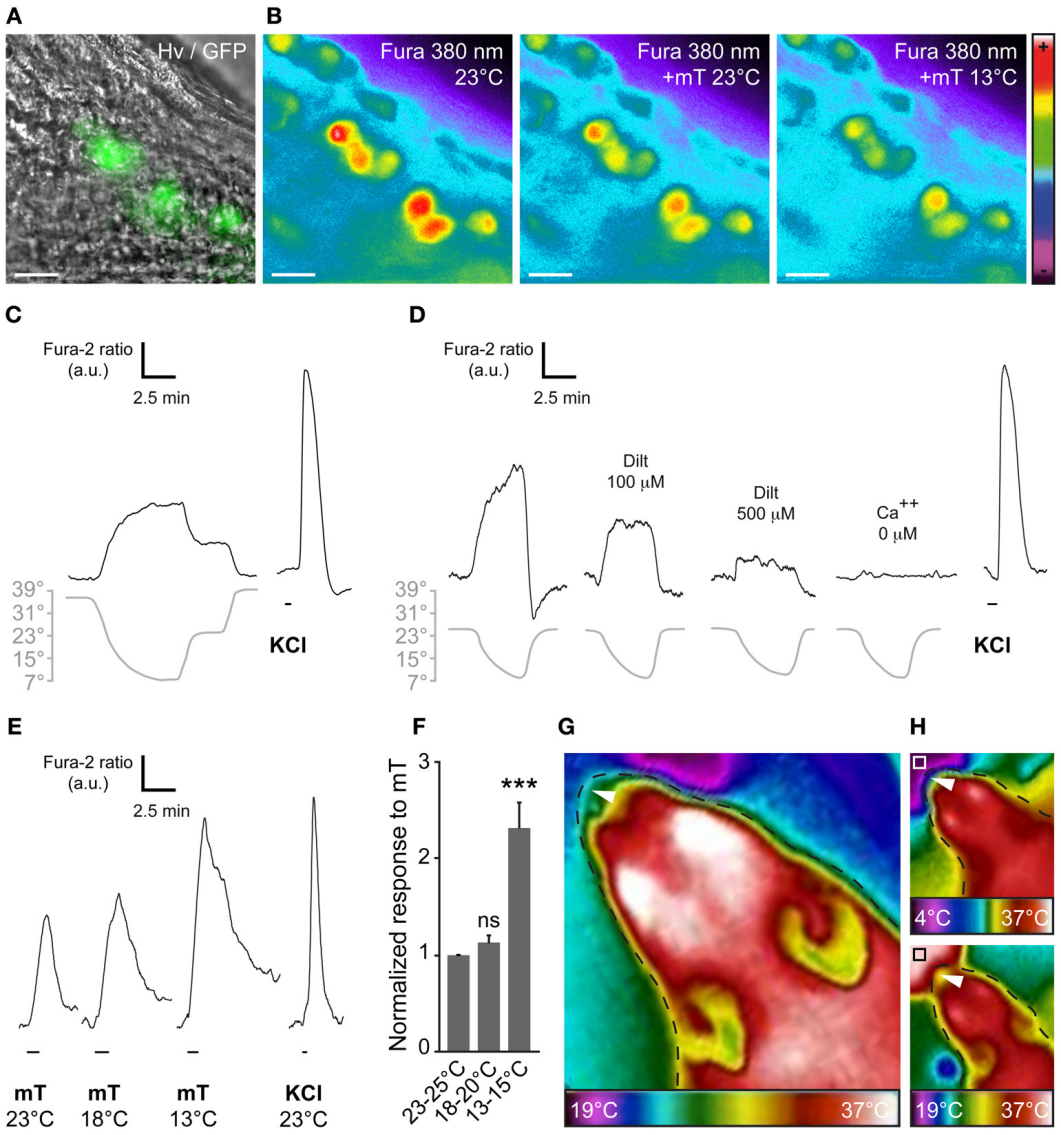
**The chemosensitivity of GG neurons is modulated by temperature variations**. **(A)** GG coronal slice from an OMP-GFP mouse, GG neurons can be observed with their intrinsic GFP fluorescence and Hoffman modulation view (Hv). **(B)** Representative Fura-2AM loaded slice observed at 380 nm in color encoded map for unbound Fura during different combination of temperature and mT perfusion. **(C–D)** Fine adjustment of the calcium level in the majority of GG neurons by temperature variations (*n* number of responding neurons/number of total neurons = 36/48; 7 mice from P1–P19). Gray lines indicate bath temperature variations. **(C)** In perfusions of 10 min, a linear intracellular adaptation of the calcium level in a single GG neuron is observed during variations in bath temperature (*n* number of tested neurons = 23). **(D)** cGMP dependence of coolness-evoked GG responses. Calcium transients could be partially inhibited by L-*cis* diltiazem [Dilt 100 μ M (32%, *n* = 5); 500 μ M (69%, *n* = 16)] and totally in calcium-free medium (*n* = 16). **(E)** Representative calcium transients induced in GG neurons by perfusion of mT (100 μ M) at different temperatures. Perfusions of KCl (20 mM) were used as viability control. **(F)** Normalized increased response to mT in function of the temperature (*n* = 25; 3 mice from P4–P23). **(G,H)** The apparent temperature of the tip of the mouse nose (GG region; white arrowheads) is dependent on the ambient temperature. Thermal view of a female mouse head (P21). **(G)** The GG region is close to the ambient temperature (25 ± 3°C). **(H)** The same mouse is observed while sniffing a cold (upper panel, white square, 4°C) or a hot (lower panel, black square, 37°C) tube. The observed temperatures of the tip of the nose are 18 ± 2°C and 29 ± 3°C, respectively. Thermal gradient scales are indicated under the mouse head pictures. Fluorescence intensity Fura-2 ratio = F340/F380 is indicated by arbitrary units (a.u.). Perfusion times are indicated by horizontal bars. Traces illustrated in **(C)**, **(D)**, and **(E)** are representative responses observed in single GG neurons for each panel. Scale bars, 20 μm in **(A)** and **(B)**. Values are expressed as mean ± s.e.m.;*t*-test, ^*^P < 0.05; ^**^P < 0.01; ^***^P < 0.001; ns, not significant.

At the single amphid neuron level, experiments have shown the modulation of neuronal responses by temperature (Biron et al., 2008; Kuhara et al., 2008). Moreover, it has been reported that cool temperature enhances the number of odorant-responsive GG neurons (Mamasuew et al., 2011a). We therefore tested the presence of a temperature-dependent chemosensitivity of GG neurons by calcium imaging on GG slices from adult and young OMP-GFP mice (Figures 4A,B). We first exposed GG neurons to continuous temperature variations of the bath perfusion (7–39°C) and observed a fine adjustment of the calcium level in the majority of GG neurons (Figure 4C; 36/48 cells). The recovery of the initial intracellular calcium level was only observed with a return to the basal temperature (Figure 4C). In an additional set of experiment, we observed that, as in amphid neurons, the coolness-evoked GG responses were also partially dependent on cGMP signaling. Indeed, L-*cis* Diltiazem (Dilt; 500 μM) was able to inhibit partially (69%) the calcium transients generated by a decrease in temperature from 25–7°C in responding neurons (*n* = 13/16) (Figure 4D; *n* = 13/13). The presence of extracellular calcium was necessary to observe the coolness-evoked response (Figure 4D; *n* = 13/13). We next evaluated the modulation of chemosensitivity of mouse GG neurons by temperature. We performed calcium imaging experiments and perfused mT at different temperatures (from 25–13°C). Interestingly, we observed in single neurons, increased mT responses with a decrease of the bath temperature (Figure 4E). This temperature modulation of the chemical response was significant when a 10°C difference was applied (Figure 4F). Thus, we here demonstrate that, not only the number of odorant-responsive GG neurons is increased by cool temperatures (Mamasuew et al., 2011a) but also the neuronal signal intensity.

Interestingly, thermal observations of the adult mouse head (Figures 4G,H) allowed us to estimate the temperature of the tip of the nose (corresponding to the GG localization). It appeared to vary with the environmental temperature, confirming the fact that GG neurons may be influenced by ambient temperature. Taken together, these results strongly support the notion of conserved multisensory modalities present in the mouse GG.

## Discussion

In *C. elegans*, the AWC class of amphid neurons are known to express different sets of proteins in parallel and convergent signaling pathways (Bargmann, 2006). These neurons possess canonical transduction cascade elements such as multi GPCRs odorant-like receptors, G_*i*_-like proteins as well as non-canonical cGMP effectors such as membrane potential receptor guanylyl cyclases or downstream proteins like CNG channels and regulatory enzymes (Bargmann, 2006). In mouse GG neurons, we report here the expression of a set of homologous proteins that probably retained similar functions. GG neurons have indeed preserved their cGMP-dependent activities (Mamasuew et al., 2010, 2011b; Hanke et al., 2013) and are implicated both in thermo- and chemo- sensing suggesting an orthologous status for the involved signaling proteins (Altenhoff and Dessimoz, 2009). The relevance of cGMP involvement for both chemo and temperature sensing in GG neurons (Mamasuew et al., 2011b; Hanke et al., 2013) was verified in our study. We also found that extracellular calcium was necessary to record both types of cellular responses but, since L-*cis* Diltiazem is not only a selective inhibitor of cyclic-nucleotide gated channels (Kraus et al., 1998; Gomora and Enyeart, 1999; Takahira et al., 2005) we cannot rule out that ion channels other than CNGA3 are implicated in the observed neuronal activations as suggested previously (Schmid et al., 2010).

Multimodalities are known to be important for the animal to sense its olfactory environment (Ma, 2010). In addition to chemical stimulation, physical stimuli can elicit responses in the different mouse olfactory subsystems. In the MOE as well as in the SO, neurons respond to both odorants and pressure using the same signaling pathway (Grosmaitre et al., 2007). Moreover, TRPM5 positive neurons found in mouse nasal cavities respond to a large variety of irritant odorants and may also be able to detect differences in temperature (Talavera et al., 2005; Lin et al., 2008a,b; Tizzano et al., 2010). Similarly to these examples it appears that GG neurons are both chemo- and thermosensitive (Brechbühl et al., 2008, 2013; Mamasuew et al., 2008, 2011a,b; Schmid et al., 2010; Hanke et al., 2013). We found that mouse GG neurons were activated by ligands of AWC amphid neurons in particular by the 2,4,5-trimethylthiazole. Moreover, GG neurons, like AWC neurons, display a chemosensitivity, that also responds to temperature variations. Nevertheless, fundamental differences exist between the behavior of amphid and GG neurons that limit comparisons. Thus, the neuronal responses in GG neurons occur during exposure to chemical cues, but they were observed afterwards in absence of chemical cues for amphid neurons (Chalasani et al., 2007). In addition, methylated thiazole structures seem to be rather more attractive for the nematode (Bargmann, 2006), while they are repulsive for the mouse (Apfelbach et al., 2005). These differences might be explained by evolutionary adaptation such as the loss or gain of unknown cellular or molecular switches (Tsunozaki et al., 2008).

The localization of the GG, close to the entry of the naris, is influenced by changes in the environmental temperature. Nevertheless, we showed here that the absence of a functional GG does not interfere with two specific pup behaviors namely thermotaxis and huddling. However, at the single neuronal level, temperature can modulate the chemosensitivity of GG neurons; a mechanism that could be implicated in the fine adjustments necessary to modulate the sensitivity of the neurons exposed to the outside environment. Interestingly, this thermo-tuning of olfactory sensing is present throughout the animal kingdom such as in nematodes (Adachi et al., 2008) or in insects (Zeiner and Tichy, 2000; Riveron et al., 2009), indicating an inherited and conserved adaptation to the environmental pressure.

Danger cues such as APs and predator scents share a structural similarity that is detected by GG neurons (Brechbühl et al., 2013). The identified mouse APs, the 2-*sec*-butyl-4,5-dihydrothiazole as well as predator scents such as the fox 2,4,5-trimethylthiazoline and the bobcat 2,6-dimethylpyrazine or other pyrazine-related cues (Mamasuew et al., 2011a) are closely related to the methyl thiazole structure that activates *C. elegans* amphid neurons. Interestingly, most of these cues are natural products of bacterial metabolism (Brown, 1979; Schellinck and Brown, 2000; Apfelbach et al., 2005; Bargmann, 2006; Zhang, 2008). The detection of these products is known to be important for odortaxis in *C. elegans* (Bargmann, 2006). In rodents, these products are, for example, generated in the guts of predators and induce upon sensing immediate attention as well as avoidance and survival strategies (Apfelbach et al., 2005). Thus, we may speculate, that the ability of an organism to detect cues from similar origin (bacterial degradation) occurs in a cluster of specialized olfactory neurons (Enjin and Suh, 2013) that has been conserved throughout evolution.

## Author contributions

Author contributions: Julien Brechbühl, Fabian Moine and Marie-Christine Broillet designed research; Julien Brechbühl, Fabian Moine, and Marie-Christine Broillet performed research; Julien Brechbühl, Fabian Moine and Marie-Christine Broillet analyzed data; and Julien Brechbühl and Marie-Christine Broillet wrote the paper.

### Conflict of interest statement

The authors declare that the research was conducted in the absence of any commercial or financial relationships that could be construed as a potential conflict of interest.

## Supplementary material

Movie 1 **Thermotaxis in a sham Ctrl mouse**. A thermal gradient is generated in the behavioral arena. Here the Ctrl mouse pup (P5) is placed in the middle of the arena and the thermotaxis behavior is recorded with a thermal camera. Temperature is indicated by the pseudocolorized scale.

Movie 2 **Thermotaxis in an Axo mouse**. A thermal gradient is generated in the behavioral arena. Here the Axo mouse pup (P5) is placed in the middle of the arena and the thermotaxis behavior is recorded with a thermal camera. Temperature is indicated by the pseudocolorized scale.

Movie 3 **Huddling in sham Ctrl mice**. Here 6 Ctrl mouse pups (P5) are placed in the middle of an arena at RT. Huddling behaviors are recorded with a thermal camera. Temperature is indicated by the pseudocolorized scale.

Movie 4 **Huddling in Axo mice**. Here 6 Axo mouse pups (P5) are placed in the middle of an arena at RT. Huddling behaviors are recorded with a thermal camera. Temperature is indicated by the pseudocolorized scale.
